# Supplementary material for: Polymorphic estrogen receptor binding site causes Cd2-dependent sex bias in the susceptibility to autoimmune diseases
Source: Nat Commun. 2021 Sep 22;12:5565. doi: 10.1038/s41467-021-25828-5 (PMC8458462; doi:10.1038/s41467-021-25828-5)
Supplement: Supplementary file 1 — Supplementary Information [file 41467_2021_25828_MOESM1_ESM.pdf]

SUPPLEMENTARY

Supplementary Figures

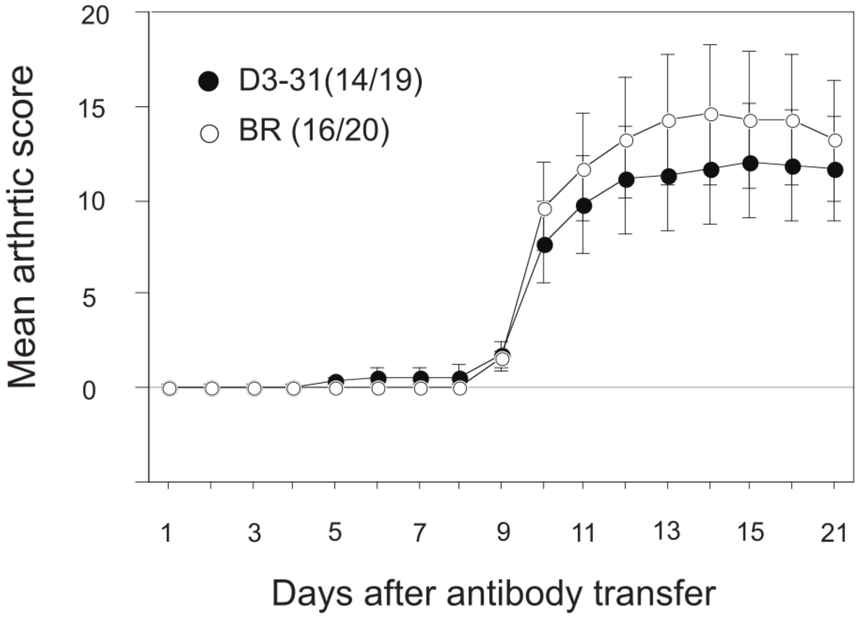

Supplementary figure 1: D3-31 mice are not protected from T cell-independent collagen type II antibody-induced arthritis (CAIA). D3-31 mice are shown in black and BR mice in white. Data is summarized as mean (SEM). Incidence and total number of mice are indicated in parenthesis. The data is representative of three independent experiments with similar results.

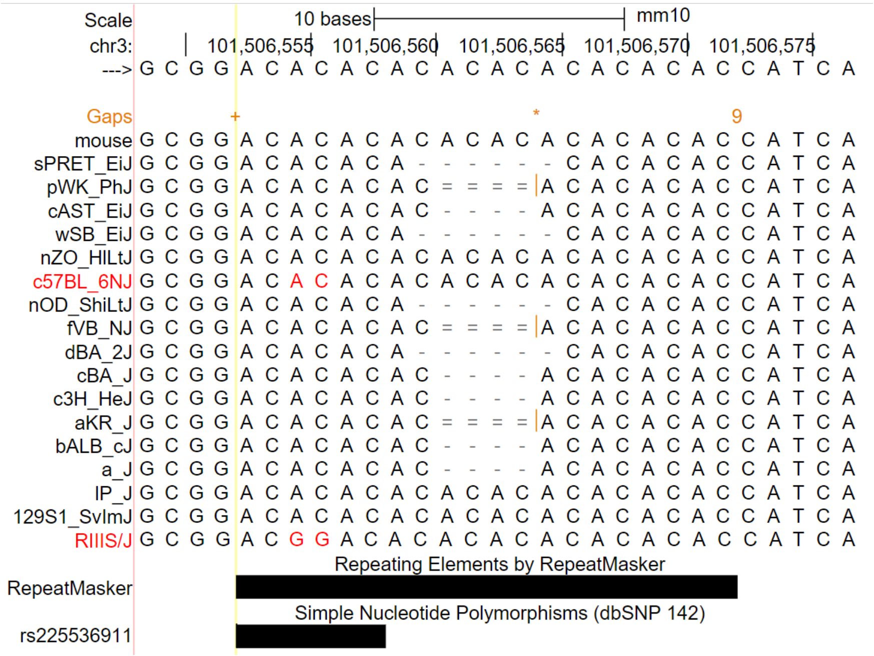

Supplementary figure 2: SNP478 lies within a highly polymorphic CA simple repeat. Sequences of common mouse inbred strains around SNP478 according to *(1)* are shown. +, start of AC repeat; 9, end of AC repeat; single line, aligned strain has no bases in the gap region; double line, aligned strain has one or more unalignable bases in the gap region.

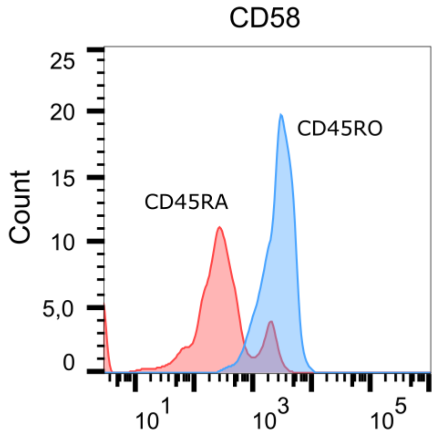

Supplementary figure 3. Flow cytometry histogram showing expression of CD58 in naïve CD45RA (red) and antigen experienced CD45RO (blue) human blood T cells. Data is representative of n = 3 independent human biological replicates. The experiment was done twice with similar results.

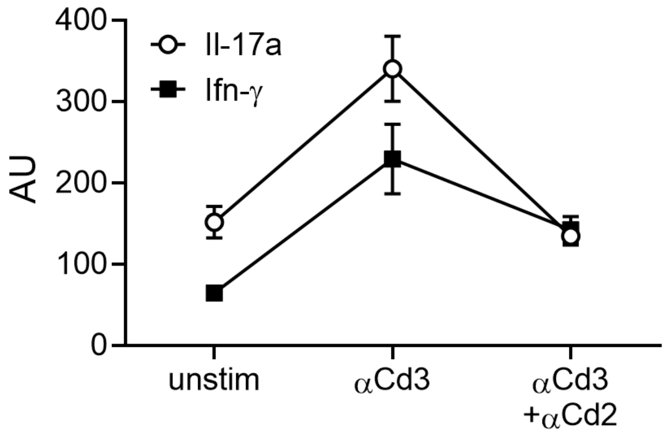

Supplementary figure 4. Complementary data to fig. 6h showing cytokine levels in the T cells cultures used for proteomic analysis. Data is summarized as mean (SEM) from n = 8 independent mouse biological replicates. The experiment was done once.

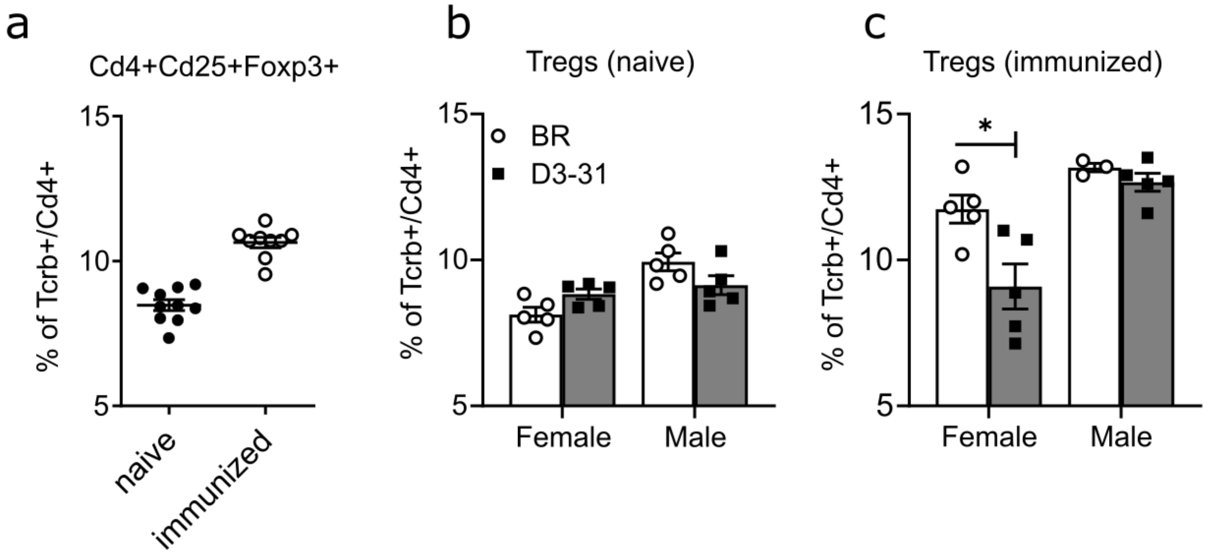

Supplementary figure 5. a) Tregs frequency in spleens from naïve compared to immunized mice. b-c) Comparison of Treg frequencies in lymph nodes from BR and D3-31, female and male, naïve (b) or immunized (c) mice. Data are summarized as mean (SEM). Each dot represents an independent biological replicate (mouse). Data is representative of two

independent experiments with similar results. Non-parametric two-tailed Mann–Whitney U test was used for statistical analysis.

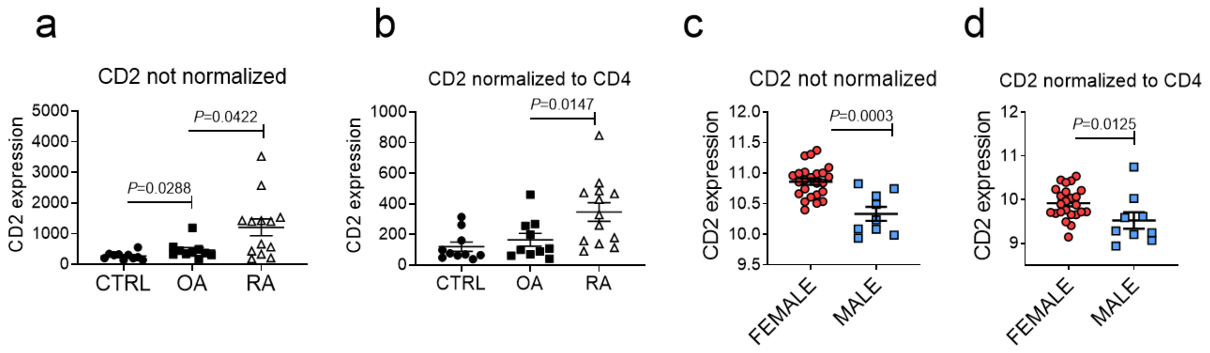

Supplementary figure 6. Complementary data to figure 7d and e showing CD2 expression without (a, c) and with (b, d) normalization to CD4 expression to account for differences in T cells numbers. Data is summarized as mean (SEM). Non-parametric two-tailed Mann–Whitney U test was used for statistical analysis.

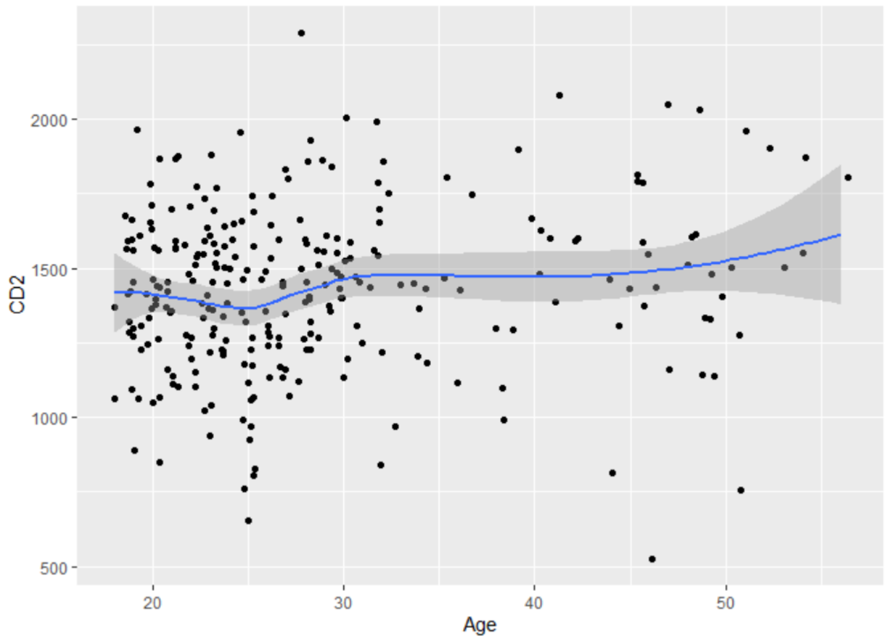

Supplementary figure 7: *CD2* expression in PBMCs from healthy women as a function of age (GEO Dataset GSE56035 *(2)*). The data is summarized as mean with 95% confidence interval. N = 452. Each dot represents one independent human biological replicate.

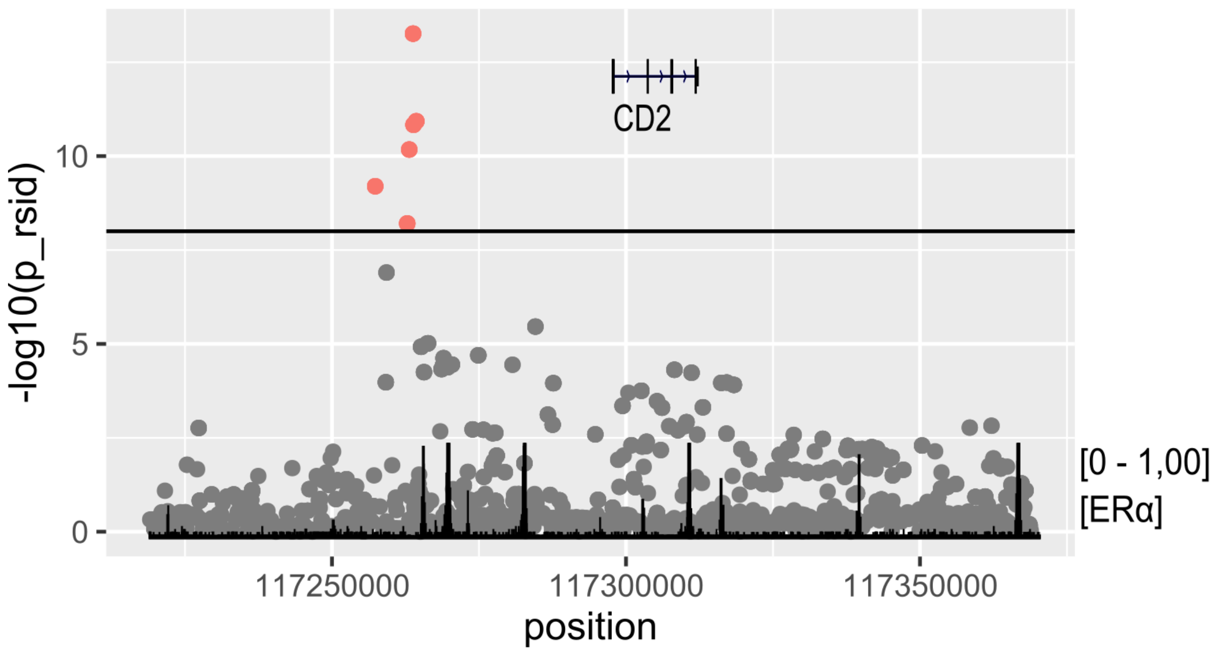

Supplementary figure 8: Meta-analysis of *CD2* locus polymorphisms associated to rheumatoid arthritis (grey/red dots) and respective ERα binding profile (black) at the bottom (extracted from *(3)*; dataset SRX1995230 and SRX3447357; Anti-ERα Sc-543; MCF-7). Summary statistics were extracted from the IEU open GWAS project *(4)* and were aggregated using Fisher's method *(5)*. Original data stem from: UK Biobank (ukb-d-M13_RHEUMA); Biobank Japan (bbj-a-72 *(6) (7)*); FinnGen Biobank (finn-a-M13_RHEUMA); and other consortia (ieu-a-832 *(8)*, ebi-a-GCST005569 *(9)*, ebi-a-GCST000679 *(10)*).

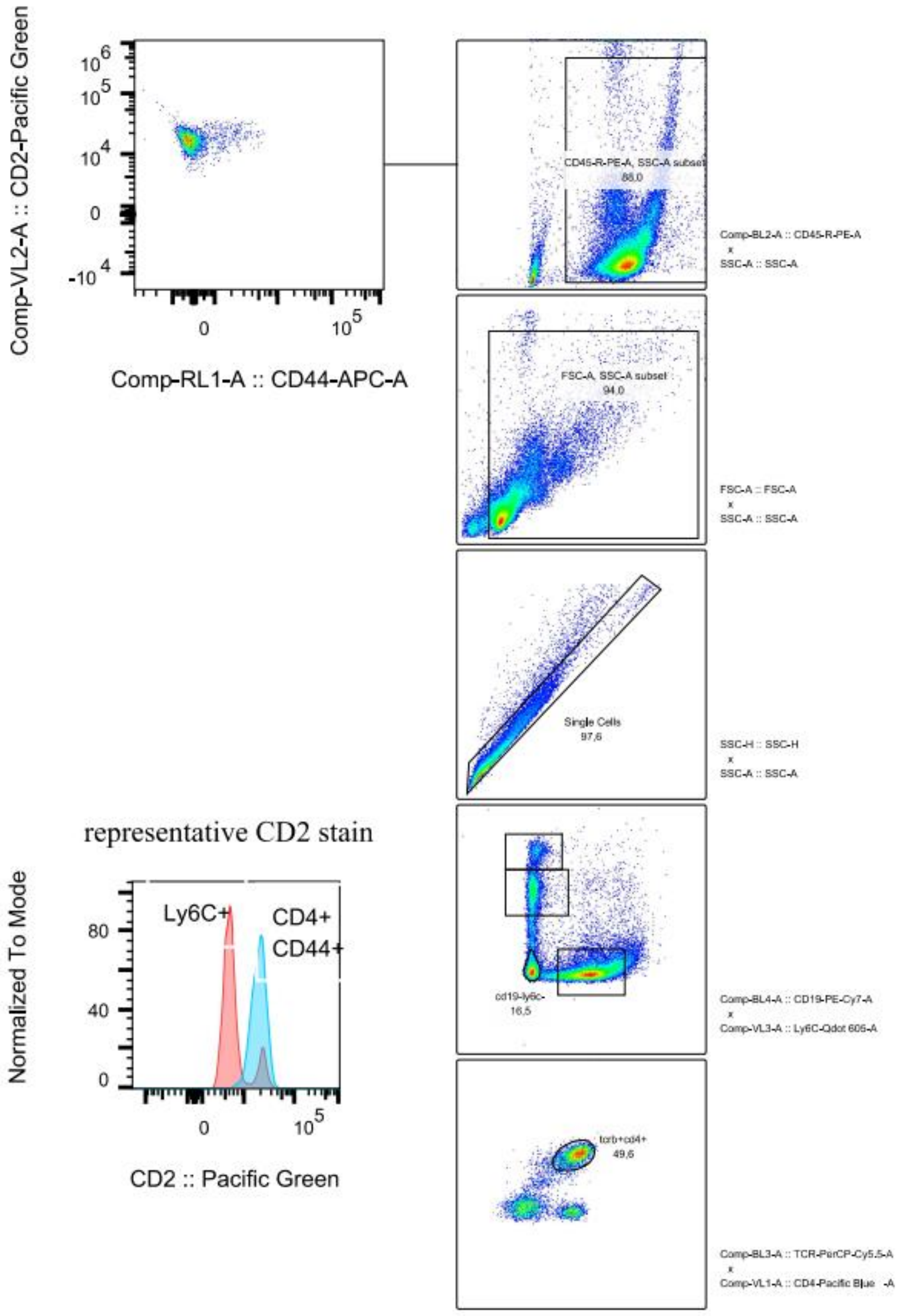

Supplementary figure 9: Flow cytometry gating strategy for fig. 5c-e.

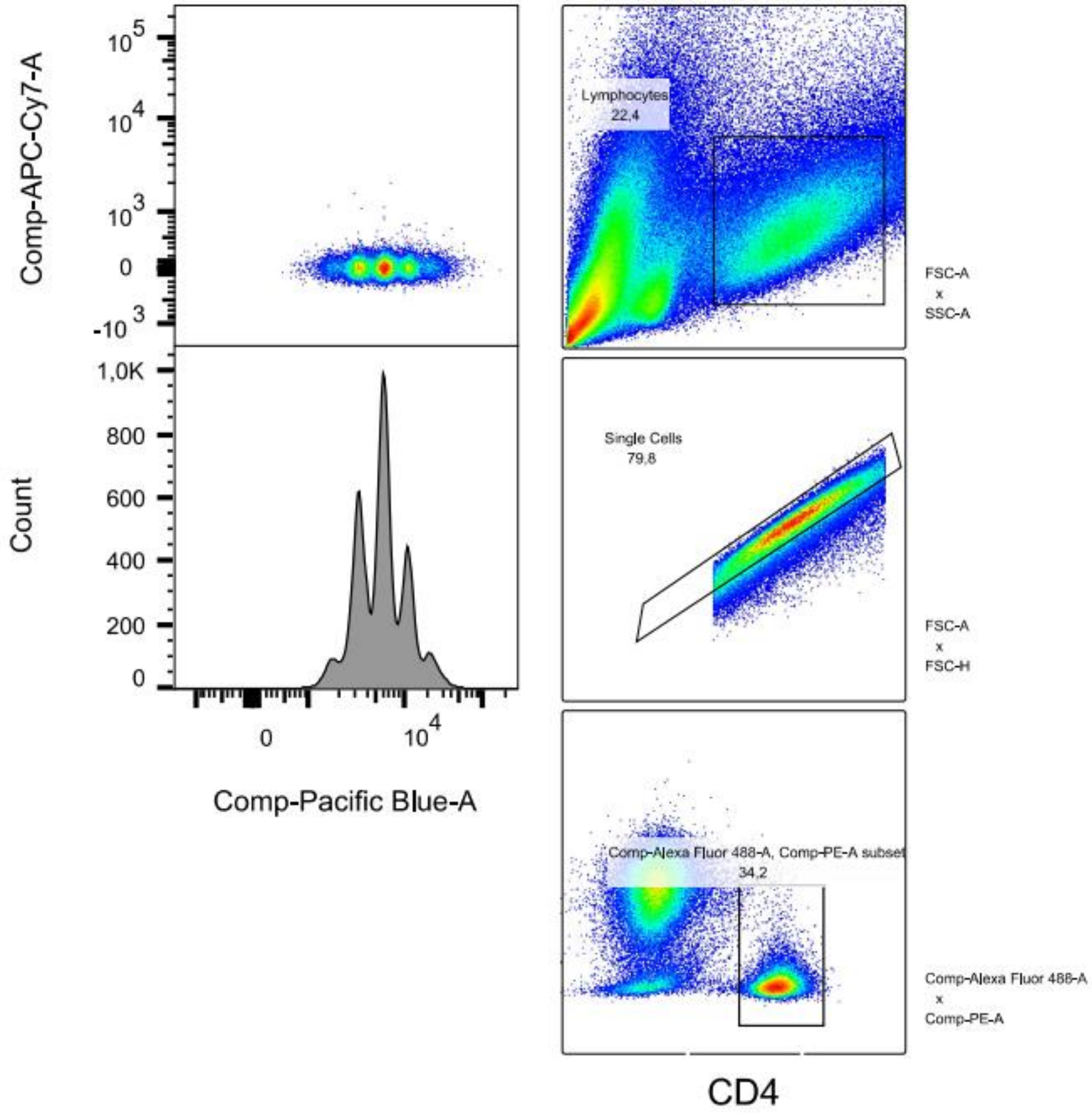

Supplementary figure 10: Flow cytometry gating strategy for fig. 6a.

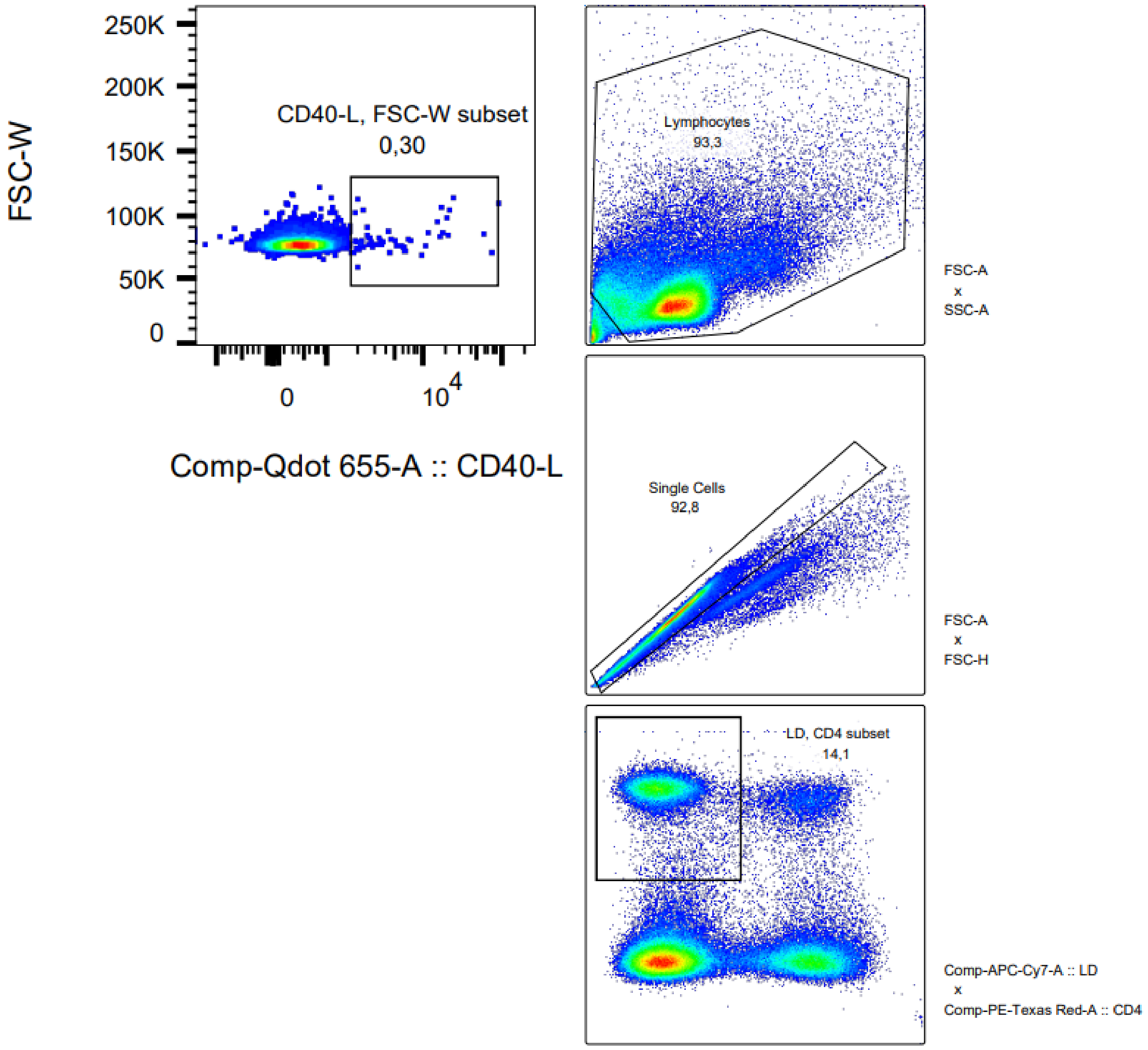

Supplementary figure 11: Flow cytometry gating strategy for fig. 6e.

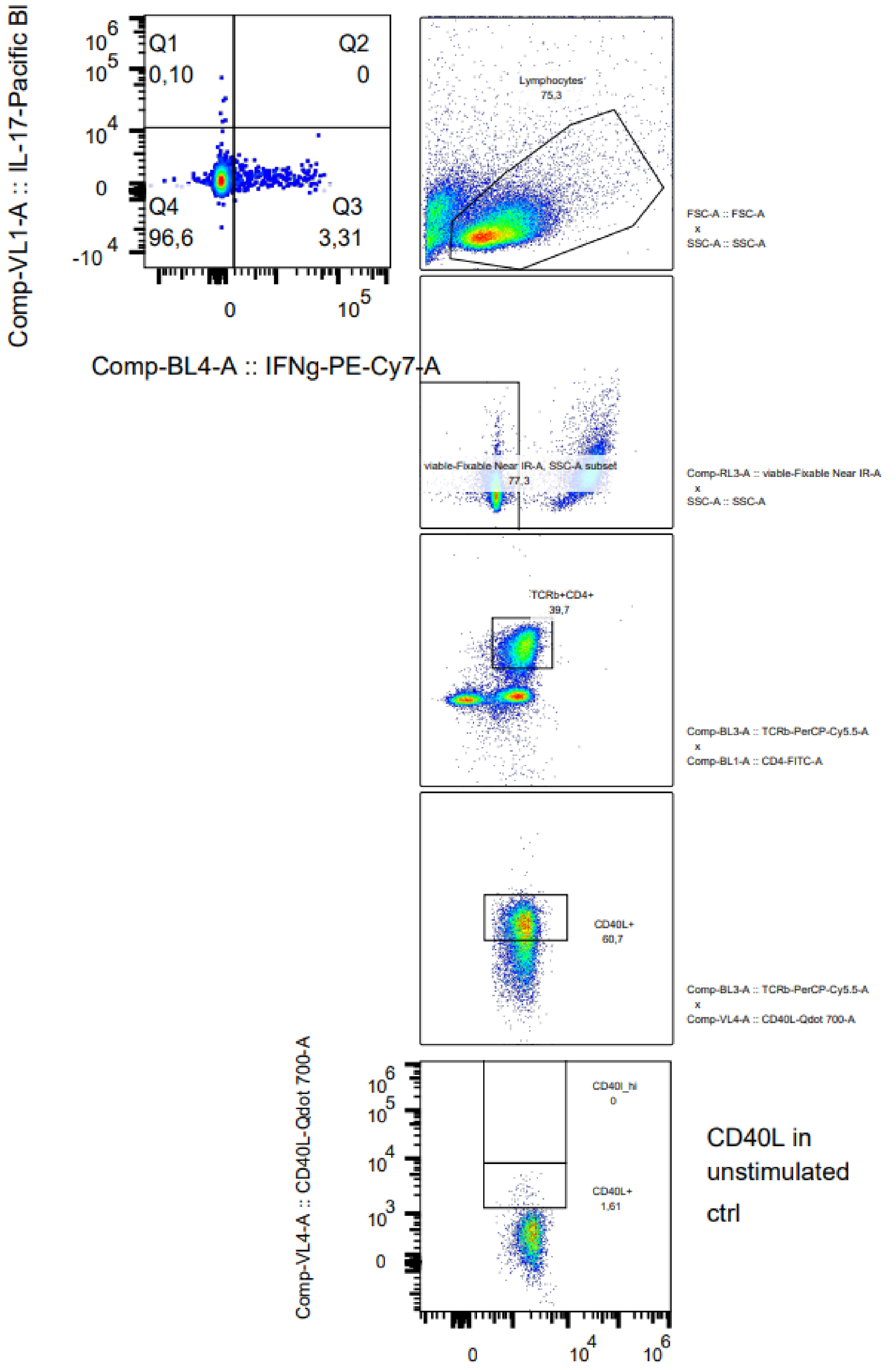

Supplementary figure 12: Flow cytometry gating strategy for fig. 6g-h.

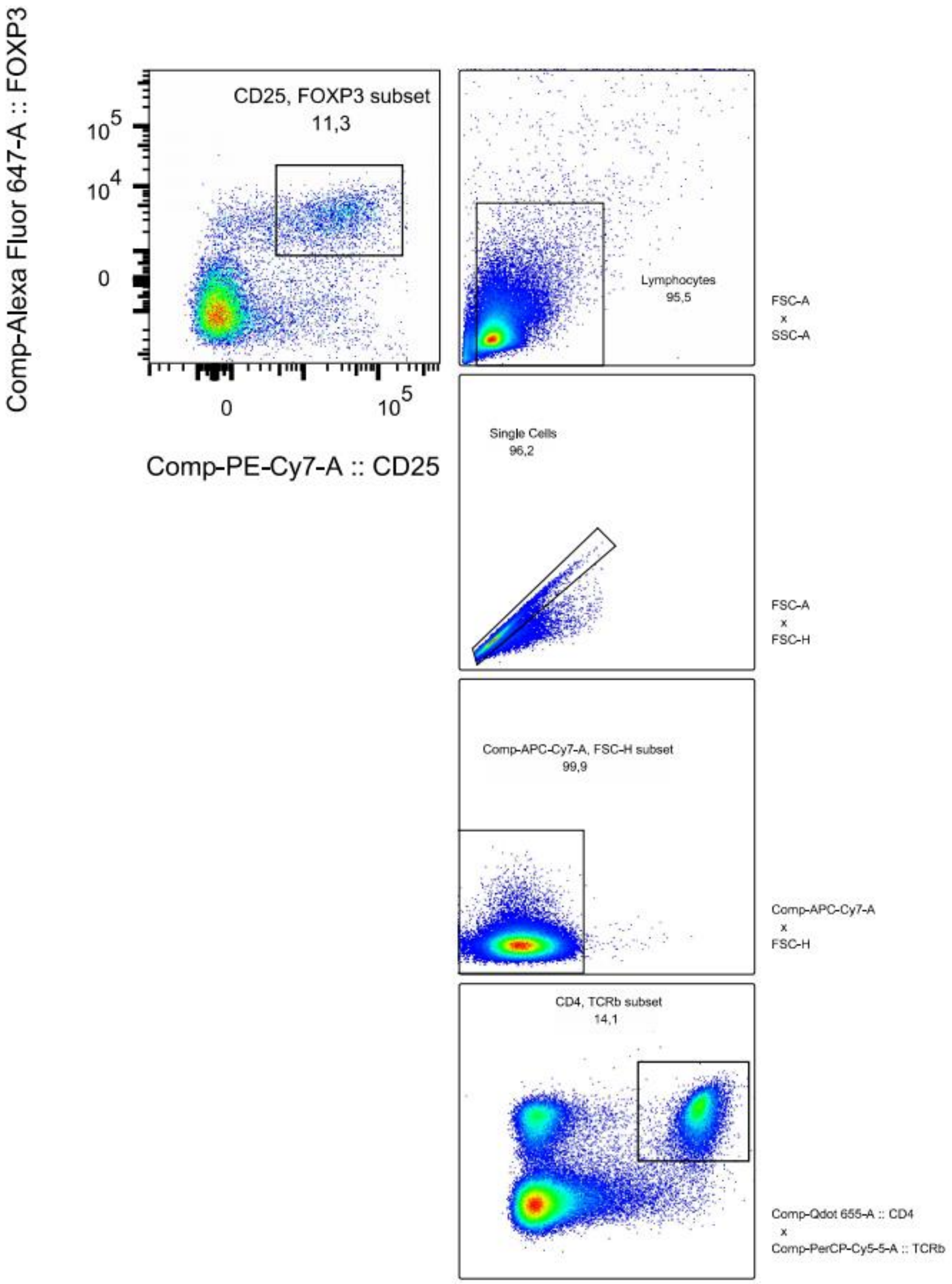

Supplementary figure 13: Flow cytometry gating strategy for fig. 6i-j.

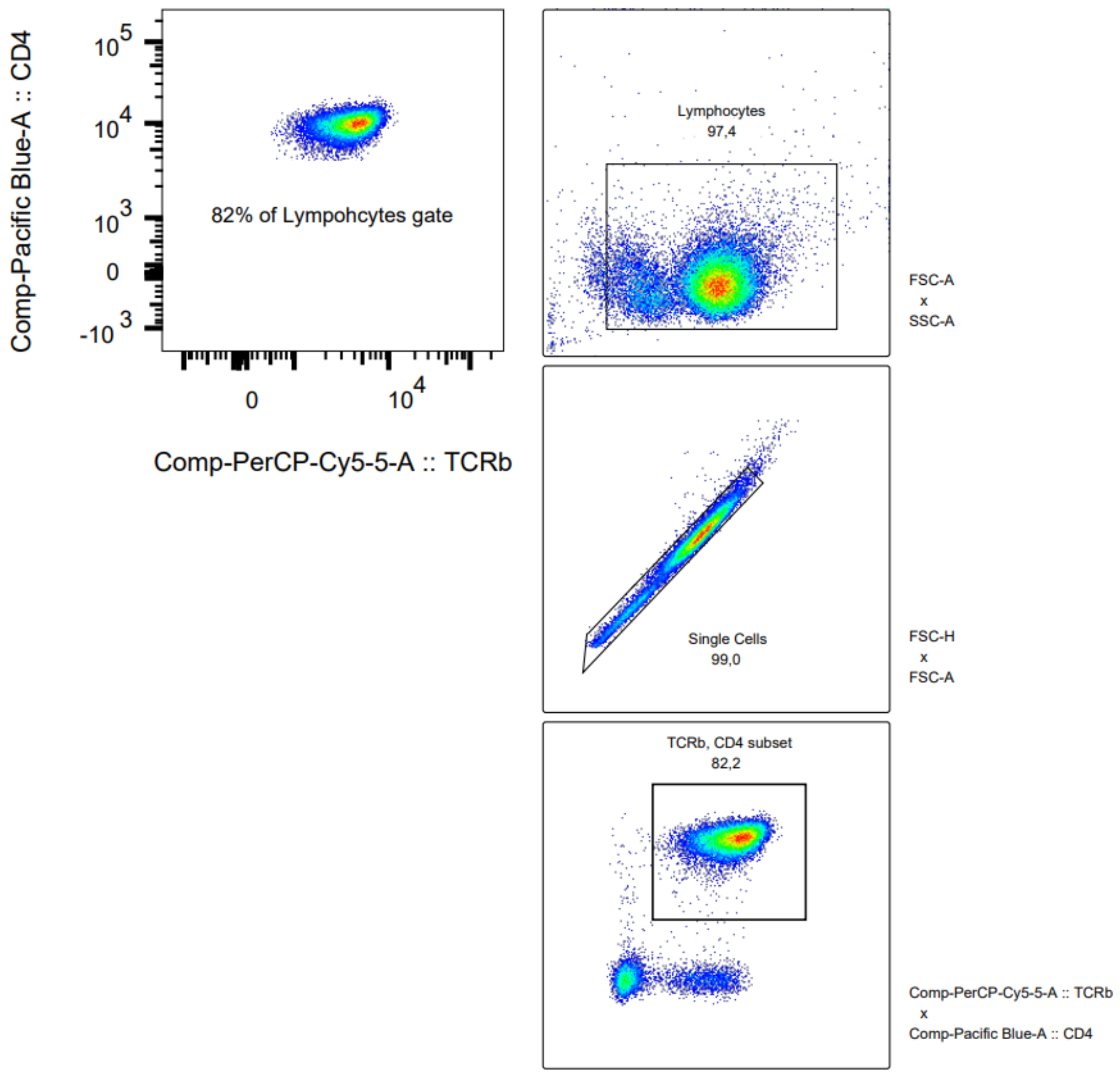

Supplementary figure 14: Purity of enriched Cd4$^+$ T cells used for proteomic analysis in fig. 6l.

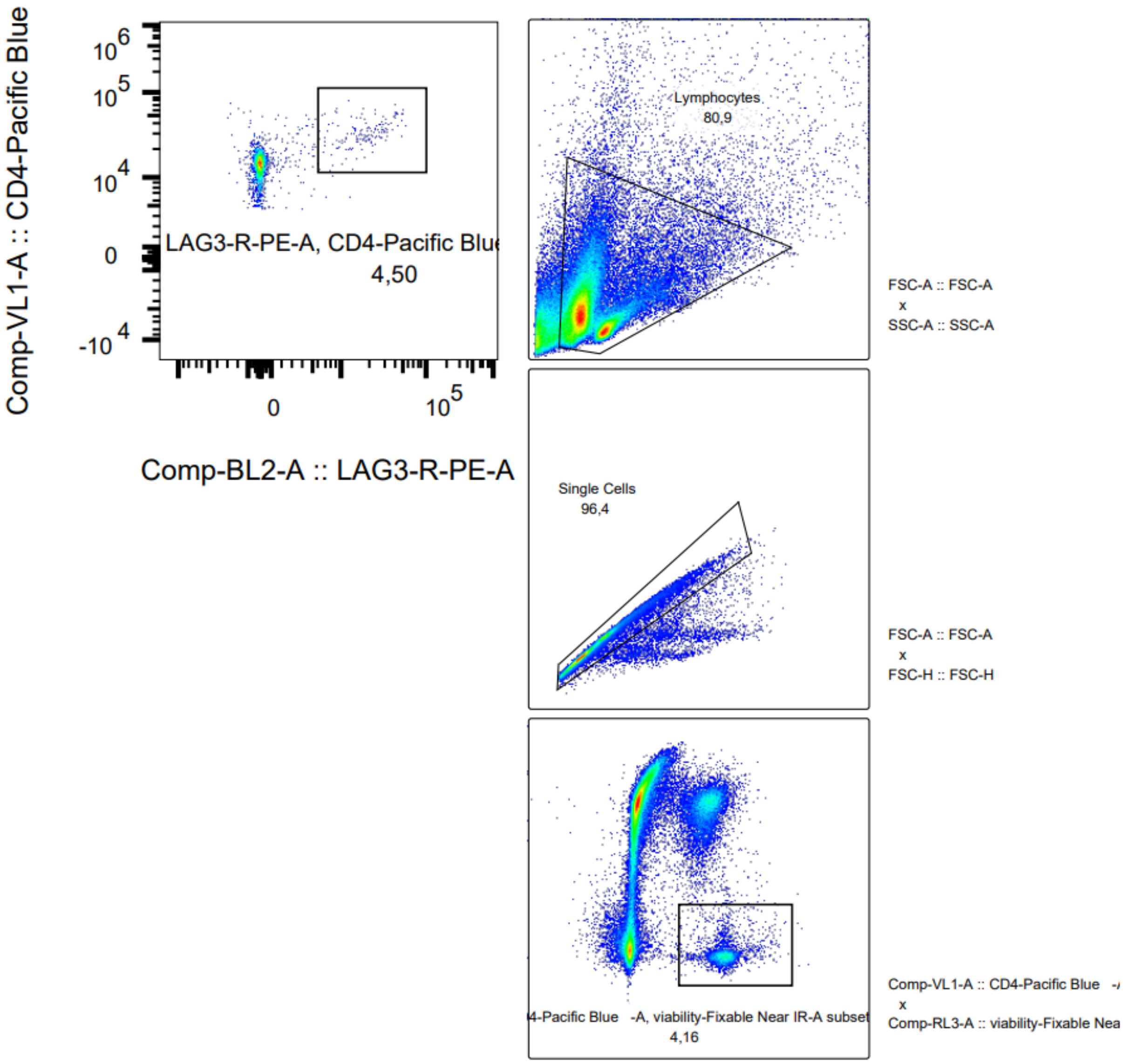

Supplementary figure 15: Flow cytometry gating strategy for fig. 6m-n.

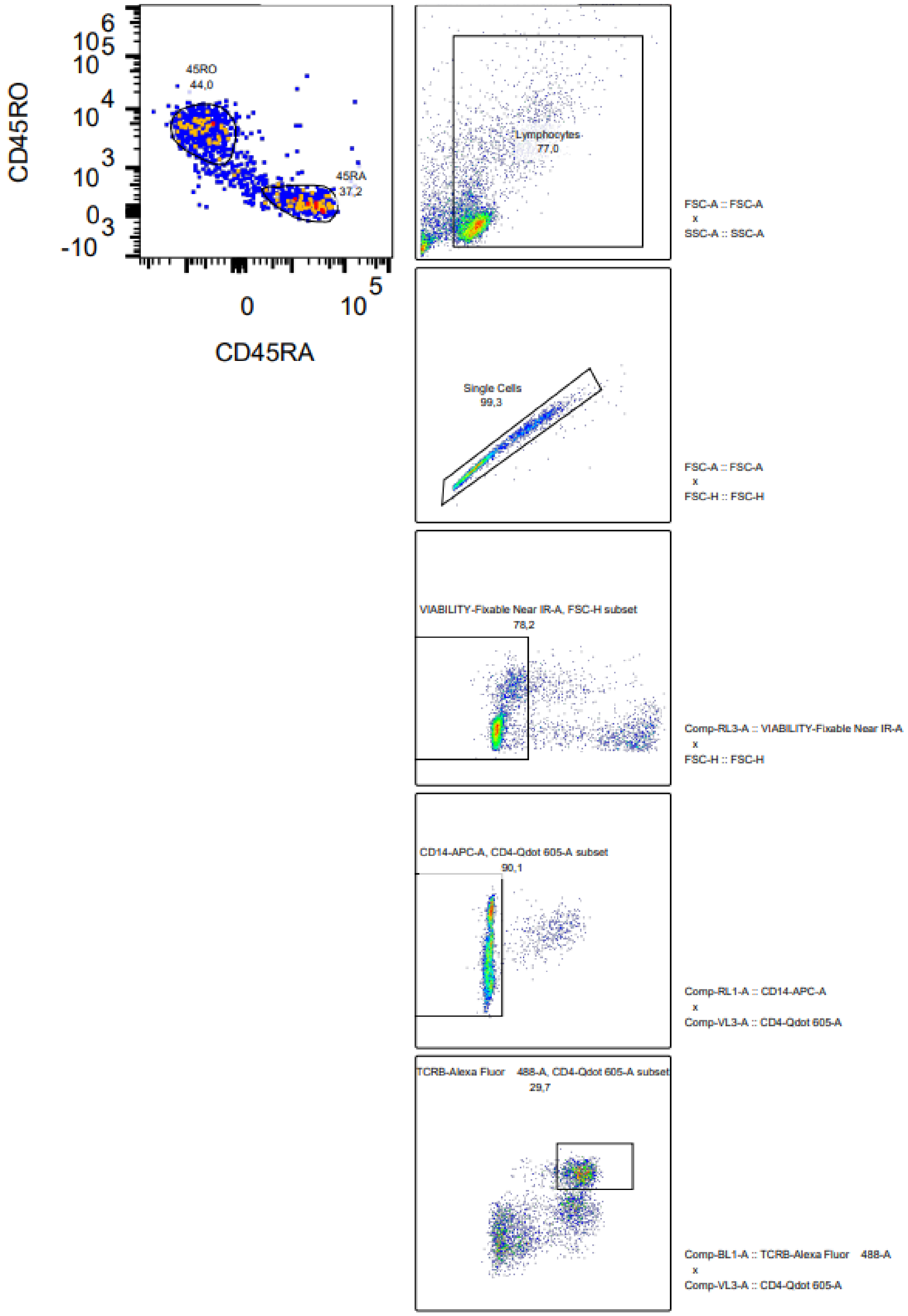

Supplementary figure 16: Flow cytometry gating strategy for fig. 7f-h.

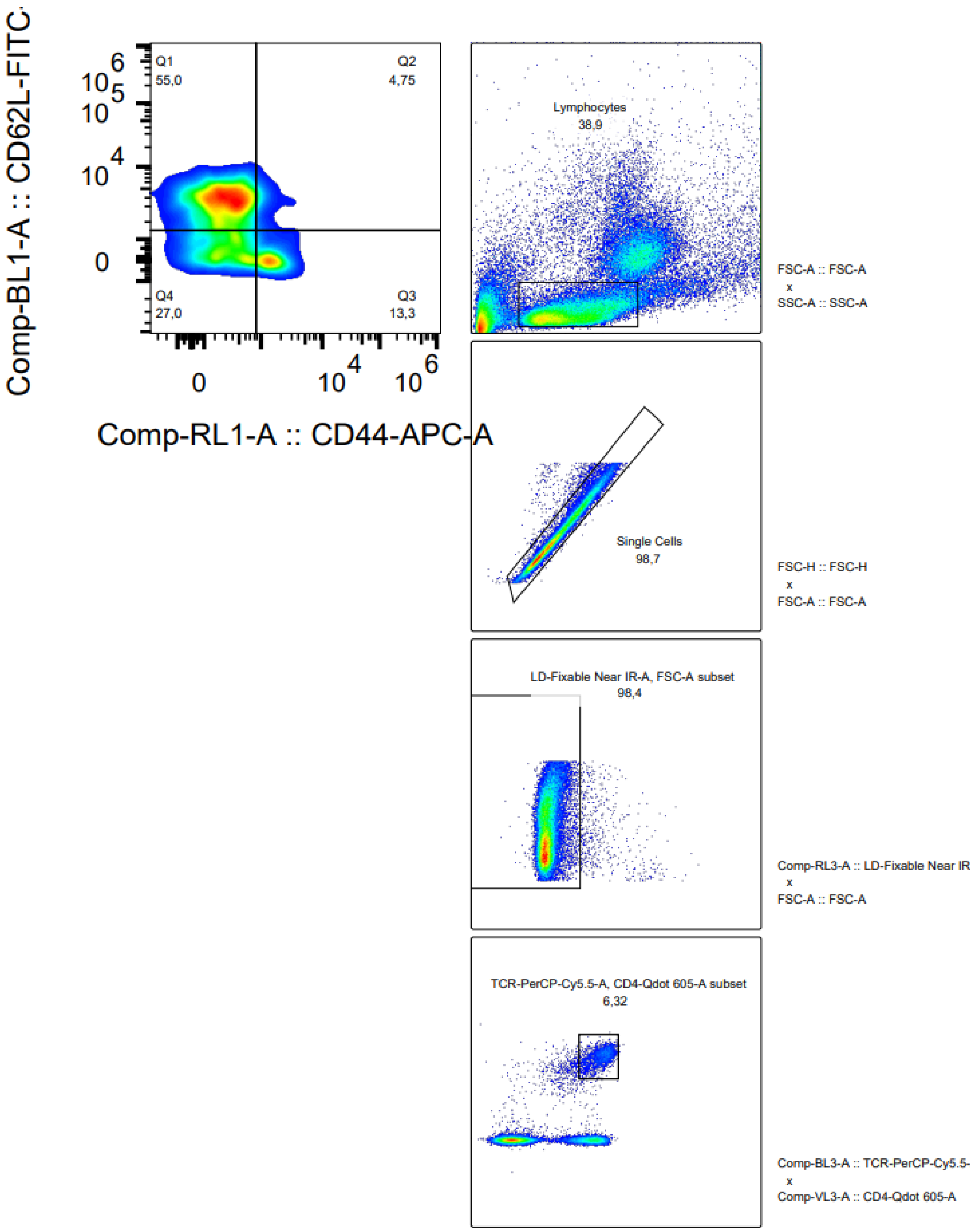

Supplementary figure 17: Flow cytometry gating strategy for fig. 8a-b.

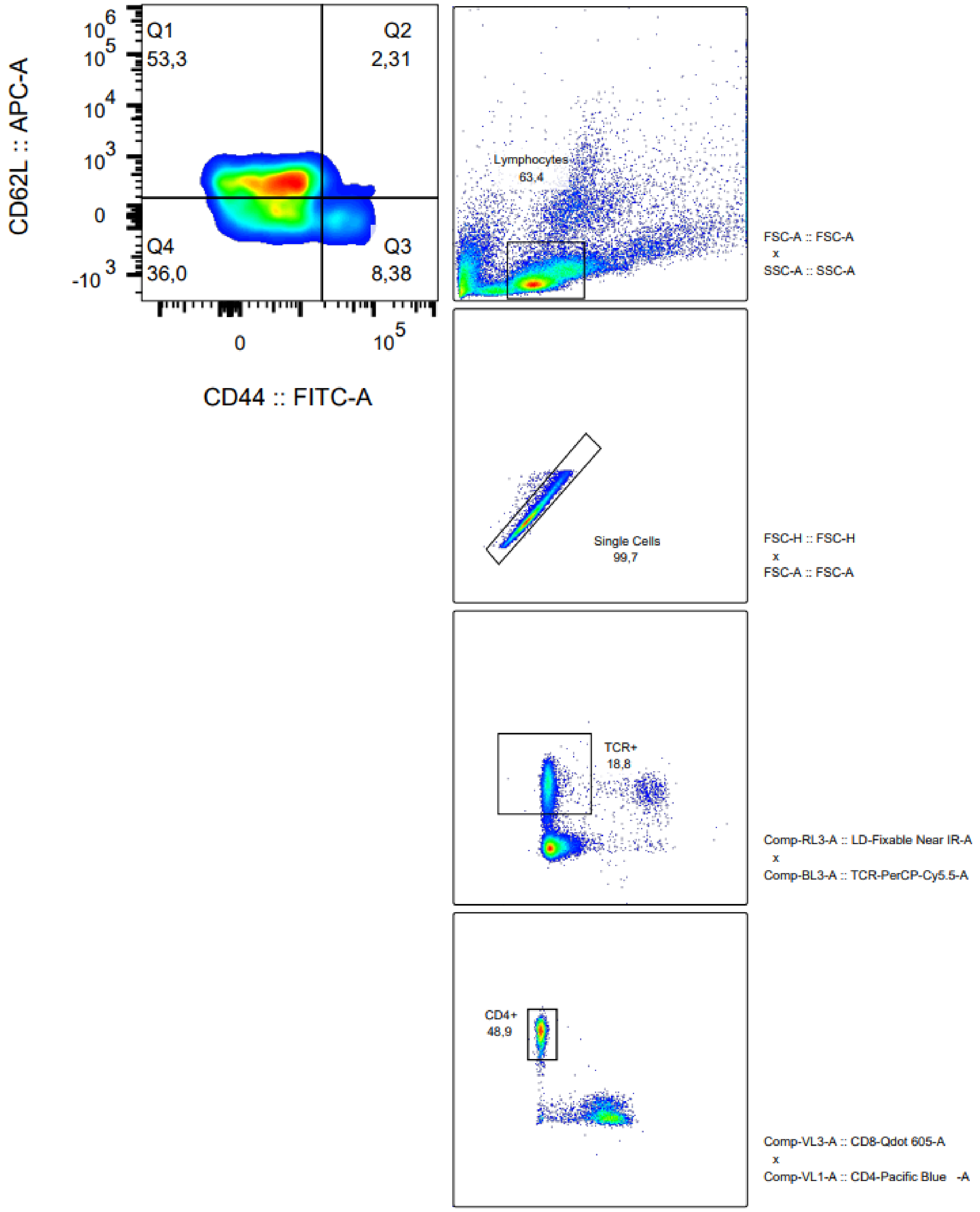

Supplementary figure 18: Flow cytometry gating strategy for fig. 8c-h.

Supplementary Table 1: List of primers used for genotyping.

Target		Sequence 5'-3'	Application
D3mit75	F	CCTGCCCTCCTTCTACCAG	genotyping
	R	GCTTAGTGGTTCCTATTGAAGTCA	
D3mf54	F	TTGTGTCTTGGCCCCTTGTT	genotyping
	R	GCTCCTAGGTGAGGCTTGAGAA	
D3mf46	F	TCCCCCAAGCATGAGAAAAC	genotyping
	R	TGGTAGCGCTTTTATTTATTCCC	
D3KV1/HRM424	F	AAGACAGGTCCCAGAACCCT	genotyping
	R	TCAGCTCTCTTCTTGACCACAC	
D3mf96	F	CCCAGAGGCTCCTCCCTAT	genotyping
	R	TCACTCTTGGTTTCGAGGAGA	
D3mf52	F	CATACATTCAGGCAAAACACTCA	genotyping
	R	TGACAGCTACGGTGTACTCACAT	
D3mf2/AA1	F	CCGATCTGATGGAGACATTTTTT	genotyping
	R	TTCTGCTGGGGTGAGATTCA	
D3mf31	F	ATGTGGCTCAATGAGAACAGCT	genotyping
	R	CCAAAAAATGGGATACCACAATG	
D3mit102	F	GGCTCGCTGGTTGGTTTTAC	genotyping
	R	GGGCTCCTACATGCATGAAT	
D3mit77	F	TCCTGCTGACACCACCAAG	genotyping
	R	AGCACCTACATTTTCCGCAA	
D3mf43	F	GTGGGGAGGTATGGGATGA	genotyping
	R	GGCTCGAACCCATGACATA	

Supplementary Table 2: List of primers used for gene expression.

Target		Sequence 5'-3'	Application
Nhlh2	F	CCACAAGCCCTTTGAGTGGG	mRNA expression
	R	CGGTGTCTTAGCGGGTGTAT	
Slc22a15	F	GCACTTGCAGGATCGATTGG	mRNA expression
	R	AGGAGCGGATGAAGTACCCT	
Cd101	F	AGCCGGCAGCAAGAGATTTT	mRNA expression
	R	GAAGCTGCGGGTTACCATCT	
Cd2	F	CCAACCTGAACGCACCATTC	mRNA expression
	R	AGGACAGACCTTTCTCTGGACA	
Atp1a1	F	TCGGGGCCATTCTTTGTTTC	mRNA expression
	R	CACCCCGAGGTACAGATCAT	
Mab21l3	F	CATTAAGGTTTTGGCCCCCA	mRNA expression
	R	GCACATTGTAGTACCGCCAG	
Igsf3	F	ATACAACTGCCGGGTGACTG	mRNA expression
	R	AGATGCTGCTTTTGAGGGGC	

Vtcn1	F	AGCTGCAGTTGCTGAACTCT	mRNA expression
	R	CTCCGGGATTCTGTTGGACC	
Actb	F	AACCGTGAAAAGATGACCCAGAT	mRNA expression
	R	GTCCATCAGAATGCCTGTGGTA	

Supplementary Table 3: List of primers used for ChIP-qCPR.

Target		Sequence 5'-3'	Application
D3KV1 1	F	GTTCTGGCCCCAGATTGTCA	ChIP-qPCR
	R	GGTTGTGCTCCTTCCTGGAG	
D3KV1 2	F	AAAGATGTGCATCTCCGCCT	ChIP-qPCR
	R	GGGACCTGTCTTTCAGGCTC	
D3KV1 3	F	ATTGGCAGTGGTGTAGCAGG	ChIP-qPCR
	R	GACAATCTGGGGCCAGAACA	
gene dessert	F	AGCTCTGCCATGAGAACACA	ChIP-qPCR (neg ctrl)
	R	TGTCCATGCCCTTCTGACAA	
gene dessert	F	TCCTCAAGACCAACTGCCAC	ChIP-qPCR (neg ctrl)
	R	GTTGCCTGTTACCACTACTGC	
Csf2ra 1	F	GACCTCCGCTAAAGTCCCAG	ChIP-qPCR (pos ctrl)
	R	TACCGCTTGTGGTCGAACTC	
Csf2ra 2	F	TGTCCTCATAGACTGGGGCA	ChIP-qPCR (pos ctrl)
	R	GGTACGCTGCTCTGTCATGT	
