# Supplementary material for: Polymorphic estrogen receptor binding site causes Cd2-dependent sex bias in the susceptibility to autoimmune diseases
Source: Nat Commun. 2021 Sep 22;12:5565. doi: 10.1038/s41467-021-25828-5 (PMC8458462; doi:10.1038/s41467-021-25828-5)
Supplement: Supplementary file 3 — Reporting Summary [file 41467_2021_25828_MOESM3_ESM.pdf]

nature research

Corresponding author(s): Rikard Holmdahl

Last updated by author(s): Aug 5, 2021

Reporting Summary

Nature Research wishes to improve the reproducibility of the work that we publish. This form provides structure for consistency and transparency in reporting. For further information on Nature Research policies, see our Editorial Policies and the Editorial Policy Checklist.

Statistics

For all statistical analyses, confirm that the following items are present in the figure legend, table legend, main text, or Methods section.

n/a	Confirmed	
☐	☒	The exact sample size (*n*) for each experimental group/condition, given as a discrete number and unit of measurement
☐	☒	A statement on whether measurements were taken from distinct samples or whether the same sample was measured repeatedly
☐	☒	The statistical test(s) used AND whether they are one- or two-sided<br>*Only common tests should be described solely by name; describe more complex techniques in the Methods section.*
☒	☐	A description of all covariates tested
☒	☐	A description of any assumptions or corrections, such as tests of normality and adjustment for multiple comparisons
☐	☒	A full description of the statistical parameters including central tendency (e.g. means) or other basic estimates (e.g. regression coefficient) AND variation (e.g. standard deviation) or associated estimates of uncertainty (e.g. confidence intervals)
☐	☒	For null hypothesis testing, the test statistic (e.g. *F*, *t*, *r*) with confidence intervals, effect sizes, degrees of freedom and *P* value noted<br>*Give P values as exact values whenever suitable.*
☒	☐	For Bayesian analysis, information on the choice of priors and Markov chain Monte Carlo settings
☒	☐	For hierarchical and complex designs, identification of the appropriate level for tests and full reporting of outcomes
☒	☐	Estimates of effect sizes (e.g. Cohen's *d*, Pearson's *r*), indicating how they were calculated

Our web collection on statistics for biologists contains articles on many of the points above.

Software and code

Policy information about availability of computer code

Data collection	Attune NxT Software v3.1; BD FACSDiva v7.0; Gen5 v2.0; CFX Manager v3.1; Gene Expression Omnibus (GEO); IEU open GWAS project.
Data analysis	Flowjo v8.8.7; Graphpad Prism v5.0-8.0; GEO2R (R v3.2.3; Biobase v2.30.0; GEOquery v2.40.0; limma v3.26.8); ShinyGEO; IGV v2.9.4; Clustal Omega v1.2.4; ieugwasr (R v4.1); PLINK v1.90b3.46; Microsoft Excel v2106

For manuscripts utilizing custom algorithms or software that are central to the research but not yet described in published literature, software must be made available to editors and reviewers. We strongly encourage code deposition in a community repository (e.g. GitHub). See the Nature Research guidelines for submitting code & software for further information.

Data

Policy information about availability of data

All manuscripts must include a data availability statement. This statement should provide the following information, where applicable:

- Accession codes, unique identifiers, or web links for publicly available datasets
- A list of figures that have associated raw data
- A description of any restrictions on data availability

The mass spectrometry proteomics data were deposited to the ProteomeXchange Consortium via the PRIDE partner repository 61 with the accession code "PXD024126 [http://proteomecentral.proteomexchange.org/cgi/GetDataset?ID=PXD024126]".

The source data for all figures (i.e. figs. 1, 2, 3, 4a, 4c-e, 5, 6, 7a and g, and 8b-c) has been deposited at Figshare under accession code "14685906 [https://doi.org/10.6084/m9.figshare.14685906.v4]".

For accessed data, accession codes and hyperlinks are provided in the following:

Fig. 4c ChIP-Atlas "SRX129062 [http://chip-atlas.org/view?id=SRX129062]"
Fig. 7b NCBI dbGaP "phs000424.v8.p2 [https://www.ncbi.nlm.nih.gov/projects/gap/cgi-bin/study.cgi?study_id=phs000424.v8.p2]"
Fig. 7c NCBI GEO "GSE45867 [https://www.ncbi.nlm.nih.gov/geo/query/acc.cgi?acc=GSE45867]";
Fig. 7d NCBI GEO "GDS5401 [https://www.ncbi.nlm.nih.gov/sites/GDSbrowser?acc=GDS5401]";
Fig. 7e NCBI GEO "GDS5363 [https://www.ncbi.nlm.nih.gov/sites/GDSbrowser?acc=GDS5363]";

Supplementary fig. 7:
NCBI GEO "GSE5603 [https://www-ncbi-nlm-nih-gov.ezproxy.u-pec.fr/geo/query/acc.cgi?acc=GSE5603]";
Supplementary fig. S8:
ChIP-Atlas "SRX1995230 [http://chip-atlas.org/view?id=SRX1995230]",
ChIP-Atlas "SRX3447357 [http://chip-atlas.org/view?id=SRX3447357]",
IEU Open GWAS "ukb-d-M13_RHEUMA [https://gwas.mrcieu.ac.uk/datasets/ukb-d-M13_RHEUMA]",
IEU Open GWAS "bbj-a-72 [https://gwas.mrcieu.ac.uk/datasets/bbj-a-72]",
IEU Open GWAS "finn-a-M13_RHEUMA [https://gwas.mrcieu.ac.uk/datasets/finn-a-M13_RHEUMA]",
IEU Open GWAS "ieu-a-832 [https://gwas.mrcieu.ac.uk/datasets/ieu-a-832]",
IEU Open GWAS "ebi-a-GCST005569 [https://gwas.mrcieu.ac.uk/datasets/ebi-a-GCST005569]",
IEU Open GWAS "ebi-a-GCST000679 [https://gwas.mrcieu.ac.uk/datasets/ebi-a-GCST000679]".

Field-specific reporting

Please select the one below that is the best fit for your research. If you are not sure, read the appropriate sections before making your selection.

☒ Life sciences ☐ Behavioural & social sciences ☐ Ecological, evolutionary & environmental sciences

For a reference copy of the document with all sections, see nature.com/documents/nr-reporting-summary-flat.pdf

Life sciences study design

All studies must disclose on these points even when the disclosure is negative.

Sample size	Sample size was chosen considering previous publications, the studied effect size, incidence of disease, and our previous experience with animal models. To resolve disease phenotypes, animal models such as CIA and EAE typically require n ≥ 10 mice per group. This is what we used in most cases. Cellular phenotypes in more controlled in vitro experiments using cell lines, mouse or human primary cells, typically require n = 5 samples for enough statistical power. We used n = 5 samples per group as we find that this gives enough statistical power while maintaining the total amount of processed samples in a feasible range. This is important because preparation of primary cells is time consuming. Some examples of publications studying animal disease models that include in vitro experiments: PMID 29513375; PMID 33087360; PMID: 33504785; PMID 32958661; PMID: 32497089.
Data exclusions	No data points were excluded from the presented data
Replication	All results were successfully replicated. Experiments were performed at least two independent times, more information is provided in the figure legends. Experiments were repeated either exactly, or in a similar setup. We exclusively used biological replicates.
Randomization	Mice of different genotypes were kept in mixed cages to avoid cage effects. Samples were also allocated randomly in culture plates to avoid plate effects.
Blinding	Animal disease model experiments were scored in a blinded manner. In vitro experiments were not blinded, as readout measurements were automated and purely quantitative, eliminating any subjective bias.

Reporting for specific materials, systems and methods

We require information from authors about some types of materials, experimental systems and methods used in many studies. Here, indicate whether each material, system or method listed is relevant to your study. If you are not sure if a list item applies to your research, read the appropriate section before selecting a response.

Materials & experimental systems

n/a	Involved in the study	
☐	☒	Antibodies
☐	☒	Eukaryotic cell lines
☒	☐	Palaeontology and archaeology
☐	☒	Animals and other organisms
☐	☒	Human research participants
☒	☐	Clinical data
☒	☐	Dual use research of concern

Methods

n/a	Involved in the study	
☒	☐	ChIP-seq
☐	☒	Flow cytometry
☒	☐	MRI-based neuroimaging

Antibodies

Antibodies used

Antibody (clone), and link with validation information follow:
BD Hamster Anti-Mouse CD3e (145-2C11), https://www.bdbiosciences.com/en-us/products/reagents/flow-cytometry-reagents/research-reagents/single-color-antibodies-ruo/purified-hamster-anti-mouse-cd3e.557306
BD Hamster Anti-Mouse TCRβ (H57-597), https://www.bdbiosciences.com/en-us/products/reagents/flow-cytometry-reagents/research-reagents/single-color-antibodies-ruo/purified-hamster-anti-mouse-tcr-chain.553167
BD Rat anti-Mouse CD4 (RM4-5), https://www.bdbiosciences.com/en-us/products/reagents/flow-cytometry-reagents/research-reagents/single-color-antibodies-ruo/purified-rat-anti-mouse-cd4.550280
BD Rat anti-Mouse CD8a (53-6.7), https://www.bdbiosciences.com/en-us/products/reagents/flow-cytometry-reagents/research-reagents/single-color-antibodies-ruo/purified-rat-anti-mouse-cd8a.550281
BD Rat anti-Mouse CD19 (1D3), https://www.bdbiosciences.com/en-us/products/reagents/flow-cytometry-reagents/research-reagents/single-color-antibodies-ruo/purified-rat-anti-mouse-cd19.553783
BD Rat anti-Mouse CD11B (M1/70), https://www.bdbiosciences.com/en-us/products/reagents/flow-cytometry-reagents/research-reagents/single-color-antibodies-ruo/purified-rat-anti-cd11b.550282
BD Hamster anti-Mouse CD11C (HL3), https://www.bdbiosciences.com/en-us/products/reagents/flow-cytometry-reagents/research-reagents/single-color-antibodies-ruo/purified-hamster-anti-mouse-cd11c.550283
BD Rat anti-Mouse FOXP3 (MF23), https://www.bdbiosciences.com/en-us/products/reagents/flow-cytometry-reagents/research-reagents/single-color-antibodies-ruo/purified-rat-anti-mouse-foxp3.560408
BD Rat anti-Mouse CD25 (7D4), https://www.bdbiosciences.com/en-us/products/reagents/flow-cytometry-reagents/research-reagents/single-color-antibodies-ruo/purified-rat-anti-mouse-cd25.558642
BD Rat anti-Mouse CD44 (IM7), https://www.bdbiosciences.com/en-us/products/reagents/flow-cytometry-reagents/research-reagents/single-color-antibodies-ruo/purified-rat-anti-mouse-cd44.553134
BD Rat anti-Mouse CD62L (MEL-14), https://www.bdbiosciences.com/en-us/products/reagents/flow-cytometry-reagents/research-reagents/single-color-antibodies-ruo/purified-rat-anti-mouse-cd62l.553152
BD Rat anti-Mouse CD2 (RM2-5), https://www.bdbiosciences.com/en-us/products/reagents/flow-cytometry-reagents/research-reagents/single-color-antibodies-ruo/purified-rat-anti-mouse-cd2.553109
BD Rat anti-Mouse LY6C (AL-21), https://www.bdbiosciences.com/en-us/products/reagents/flow-cytometry-reagents/research-reagents/single-color-antibodies-ruo/purified-rat-anti-mouse-ly-6c.560595
BD Rat anti-Mouse LAG-3 (C9B7W), https://www.bdbiosciences.com/en-us/products/reagents/flow-cytometry-reagents/research-reagents/single-color-antibodies-ruo/purified-rat-anti-mouse-cd223.552380
BD Hamster anti-Mouse CD40L (MR1), https://www.bdbiosciences.com/en-us/products/reagents/flow-cytometry-reagents/research-reagents/single-color-antibodies-ruo/purified-hamster-anti-mouse-cd154-cd40-ligand.745722
BD Rat anti-Mouse IFN-γ (R46A2), https://www.bdbiosciences.com/en-us/products/reagents/immunoassay-reagents/purified-rat-anti-mouse-ifn.551216
Mabtech Rat anti-Mouse IFN-γ (R46A2), https://www.mabtech.com/products/anti-mouse-ifn-gamma-antibody-r4-6a2-biotinylated-3321-6
Mabtech Rat anti-mouse IFN-γ mAb (AN18), https://www.mabtech.com/products/anti-mouse-ifn-gamma-antibody-an18-purified-3321-3
BD Rat anti-Mouse IL-17A (TC11-18H10.1), https://www.bdbiosciences.com/en-us/products/reagents/flow-cytometry-reagents/research-reagents/single-color-antibodies-ruo/purified-rat-anti-mouse-il-17a.559501
BD Rat anti-Mouse IL-17A (TC11-8H4), https://www.bdbiosciences.com/en-us/products/reagents/immunoassay-reagents/elisa/biotin-rat-anti-mouse-il-17a.555067

BD Mouse anti-Human CD45 (clone: HI30), https://www.bdbiosciences.com/en-us/products/reagents/flow-cytometry-reagents/research-reagents/single-color-antibodies-ruo/purified-mouse-anti-human-cd45.555483
BD Mouse anti-Human CD2 (RPA-2,10), https://www.bdbiosciences.com/en-us/products/reagents/flow-cytometry-reagents/research-reagents/single-color-antibodies-ruo/purified-mouse-anti-human-cd2.555324
BD Mouse anti-Human TCRB (IP26), https://www.bdbiosciences.com/en-us/products/reagents/flow-cytometry-reagents/research-reagents/single-color-antibodies-ruo/purified-mouse-anti-human-tcr.564728
BD Mouse anti-Human CD4 (OKT4), https://www.bdbiosciences.com/en-us/products/reagents/flow-cytometry-reagents/research-reagents/single-color-antibodies-ruo/pe-mouse-anti-human-cd4.566680
BD Mouse anti-Human CD45RA (HI100), https://www.bdbiosciences.com/en-us/products/reagents/flow-cytometry-reagents/research-reagents/single-color-antibodies-ruo/purified-mouse-anti-human-cd45ra.563963
BD Mouse anti-Human CD45RO (UCHL1), https://www.bdbiosciences.com/en-us/products/reagents/flow-cytometry-reagents/research-reagents/single-color-antibodies-ruo/purified-mouse-anti-human-cd45ro.555493
Abcam Rabbit anti-Mouse Erα, https://www.abcam.com/estrogen-receptor-alpha-antibody-e115-chip-grade-ab32063.html

BD Rat anti-Mouse IL-2 (JES6-IA12), https://www.bdbiosciences.com/en-us/products/reagents/flow-cytometry-reagents/research-reagents/single-color-antibodies-ruo/purified-rat-anti-mouse-il-2.554424
BD Hamster anti-Mouse CD3 (500A2), https://www.bdbiosciences.com/en-us/products/reagents/flow-cytometry-reagents/research-reagents/single-color-antibodies-ruo/purified-hamster-anti-mouse-cd3e.553238
BD Hamster anti-Mouse CD28 (37.51), https://www.bdbiosciences.com/en-us/products/reagents/flow-cytometry-reagents/research-reagents/single-color-antibodies-ruo/purified-hamster-anti-mouse-cd28.557393
In house Rat anti-Mouse (JES6-5H4), https://www.mabtech.com/products/anti-mouse-il-2-antibody-5h4-biotinylated-3441-6
In house Rat anti-Mouse CD16/CD32 (2.4G2), https://www.bdbiosciences.com/en-us/products/reagents/flow-cytometry-reagents/research-reagents/single-color-antibodies-ruo/purified-rat-anti-mouse-cd16-cd32-mouse-bd-fc-block.553142

Validation

All antibodies have been validated in peer-reviewed publications or by the manufacturer. Information on the validation of the idividual antibodies can be found under the links provided in the field above.

Eukaryotic cell lines

Policy information about cell lines

Cell line source(s)	MCF-7 were originally obtained from ATCC
Authentication	Cell lines were not authenticated
Mycoplasma contamination	Cell lines were not tested for mycoplasma
Commonly misidentified lines (See ICLAC register)	No commonly misidentified cell lines were used.

Animals and other organisms

Policy information about studies involving animals; ARRIVE guidelines recommended for reporting animal research

Laboratory animals	Species: Mus Musculus; Strains: C57BL/10J and RIIIS/J; Sex: female and male, Age: 12 weeks. Mice were kept under specific pathogen free (SPF) conditions following FELASA II guidelines. Animals were housed in individually ventilated cages containing wood shavings in a climate-controlled environment (21-23°C, 40-50% humidity) with a 12 h light-dark cycle, fed with standard chow and water ad libitum. All the experiments were performed with age-, sex- and cage-matched mice and all the genetic experiments were performed with littermate controls.
Wild animals	study did not involve wild animals
Field-collected samples	study did not involve field-collected samples
Ethics oversight	Jordbruksverket, regional ethic committee, Stockholm, Sweden.

Note that full information on the approval of the study protocol must also be provided in the manuscript.

Human research participants

Policy information about studies involving human research participants

Population characteristics	healthy volunteers, age: 28-40, gender: mixed female and male, no genotypic information, no known diagnosis
Recruitment	Colleagues were recruited on a voluntary basis. One important bias may be the age of the participants. The subjects tested here are middle aged, and cells from significantly younger or older subjects may exhibit a different response to estrogens due to differences in hormonal levels or hormone receptor expression. Further, one should consider that we tested healthy subjects. The behavior from healthy cells may deviate from that of cells in an inflammatory environment.
Ethics oversight	Etikprövningsmyndigheten, Swedish ethical Review authority, Uppsala, Sweden.

Note that full information on the approval of the study protocol must also be provided in the manuscript.

Flow Cytometry

Plots

Confirm that:

- [x] The axis labels state the marker and fluorochrome used (e.g. CD4-FITC).
- [x] The axis scales are clearly visible. Include numbers along axes only for bottom left plot of group (a 'group' is an analysis of identical markers).
- [x] All plots are contour plots with outliers or pseudocolor plots.
- [x] A numerical value for number of cells or percentage (with statistics) is provided.

Methodology

Sample preparation	Mouse organs were harvested directly after termination of mice by CO2 and kept in PBS until mechanic dissociation on cell strainers. Cell suspensions were washed in PBS and red blood cells lysed if applicable. Thereafter cells were counted, and typically 1M cells were plated for staining. For more details on cell preparation and flow cytometry staining protocol please refer to Materials and Methods. Human PBMCs were prepared from blood of healthy donors using SepMate (Stemcell Technologies) as stated in Materials and Methods.
Instrument	LSR II (BD), Attune NxT (Thermo Scientific)
Software	BD FACSDiva v7.0 , Attune NxT Software v3.1, FlowJo v8.8.7

Cell population abundance	Only applicable to proteomic analysis in fig. 6h, the abundance of CD4+ cells afer enrichment was 82%. Cell frequencies in other experiments are stated in FACS plots.
Gating strategy	A typical gating strategy is as follows: Lymphocytes > singlets > viability > relevant markers (e.g. TCRb+ > CD4+ > CD44+). Figure exemplifying gating strategy will be added if manuscript is considered for revision.

☒ Tick this box to confirm that a figure exemplifying the gating strategy is provided in the Supplementary Information.
